# Survey of the genome of *Pogostemon cablin* provides insights into its evolutionary history and sesquiterpenoid biosynthesis

**DOI:** 10.1038/srep26405

**Published:** 2016-05-20

**Authors:** Yang He, Hongtao Xiao, Cao Deng, Liang Xiong, Hu Nie, Cheng Peng

**Affiliations:** 1 State Key Laboratory Breeding Base of Systematic Research, Development and Utilization of Chinese Medicine Resources, Chengdu University of Traditional Chinese Medicine, Chengdu 610075, China; 2 School of Medicine, University of Electronic Science and Technology of China, Chengdu 610072, China; 3 Department of Pharmacy, Hospital of the University of Electronic Science and Technology of China and Sichuan Provincial People’s Hospital, Chengdu 610072, China; 4 DNA Stories Bioinformatics Center, Chengdu 610065, China

## Abstract

*Pogostemon cablin* (Blanco) Benth. (Patchouli) is an important traditional Chinese medicinal plant that has both essential oil value and a broad range of therapeutic effects. Here we report the first *de novo* assembled 1.15-Gb draft genome sequence for *P. cablin* from next-generation sequencing technology. Our assembly, with a misassembly rate of <4 bp per 100 kb, is ~73% of the predicted genome size (1.57 Gb). Analysis of whole-genome sequences identified 3,147,333 heterozygous single-nucleotide polymorphisms and 490,407 insertions and deletions, giving an estimated heterozygosity rate of 0.274%. A comprehensive annotation pipeline indicated that repetitive sequences make up 58.55% of the assemblies, and that there are estimated 45,020 genes. Comparative genomics analysis showed that the Phrymaceae and Lamiaceae family split ~62.80 Mya, and the divergence between patchouli and sesame occurred ~52.42 Mya, implying a potentially shared recent whole-genome duplication event. Analysis of gene homologs involved in sesquiterpenoid biosynthesis showed that patchouli contains key genes involved in more sesquiterpenoid types and has more copies of genes for each sesquiterpenoid type than several other related plant species. The patchouli genome will facilitate future research on secondary metabolic pathways and their regulation as well as potential selective breeding of patchouli.

Lamiaceae, the mint family of flowering plants, has broad distribution and, with more than 7000 species, and it is the largest family of the order Lamiales. Plants from this family are valued for their flavor, fragrance, and medicinal properties. *Pogostemon* is a large genus from this family, and one of the best-known members of this genus is patchouli, *Pogostemon cablin*. Patchouli is an annual herb native to the Philippines^1^ and has been widely cultivated in tropical and subtropical areas of Asia^2^ owing to high demand for its essential oil and precious material for traditional medicine in China. The patchouli alcohol, which is abundant in the plant leaves, is an important ingredient for perfumes, incense, soaps and cosmetic products^3^,^4^. Patchouli is also an essential Chinese medicinal plant^5^ recorded in Chinese Pharmacopoeia^6^ for its therapeutic roles in heat and dampness elimination, nerve smoothness and fatigue alleviation. Patchouli is also a component of some proprietary Chinese medicines that could be used to treat indigestion, headache, and fever^6^.

Chemical and pharmacological studies of *P. cablin* over the last few decades indicate that patchouli contains >40 major components, including mono- and sesqui-terpenoids^7^, triterpenoids and steroids^8^, flavonoids^9^, and alkaloids and phenylpropanoid glycosides^10^. The constituents exhibit marked activities, such as antibacterial^11^,^12^, anti-influenza virus^13^, anti-inflammatory^14^, cytotoxic^15^, antimutagenic^9^, anti-PAF–induced platelet aggregation^16^, insecticidal, and hepatoprotective activities^17^. Among these components, sesquiterpenoids and flavonoids are most abundant^18^. However, although some genes involved in terpenoid and flavonoid biosynthesis have been isolated and functionally characterized^18^,^19^, the global terpenoid and flavonoid biosynthesis pathways in *P. cablin* remain to be fully characterized.

In this genomic era, deciphering genomes of medicinal plants is of great importance in understanding and improving these poorly investigated species and will enable insights into the biochemistry and evolution of genes responsible for secondary metabolism biosynthetic pathways. Recent advances in genome sequencing technologies and assembly algorithms have resulted in the generation of genome sequences for a wide range of plant species. However, very few studies have examined the genomes of medicinal plants^20^. Currently, only the genomes of two medicinal mushrooms (*Ganoderma lucidum*^21^ and *G. sinense*^22^) and two medicinal flowering plants (*Dendrobium officinale*^23^ and *Catharanthus roseus*^24^) have been reported, and these studies highlight that genomic data could play an important role in investigating secondary metabolic pathways.

Despite the prominent roles of patchouli in both industrial and traditional Chinese medicine, our understanding of its biology is limited by a lack of genomic resources. To set up the foundation for genomic studies of patchouli, we assembled the draft genome of *P. cablin*, which is also the first draft genome of Lamiaceae. Although cost and time restraints prohibited assembly of the complete genome, this draft genome does capture the vast majority of patchouli genomic regions. The availability of this genome sequence not only is an invaluable resource for elucidating evolutionary processes in the Lamiaceae lineage but also provides insight into the underlying molecular mechanism of the sesquiterpenoid biosynthesis pathway in patchouli. This genomic resource will serve as a foundation for further investigation of patchouli biology and for the selection of cultivars with improved medicinal and pharmaceutical traits.

## Results and Discussion

### Genome sequencing and quality control

To obtain sufficient quantities of nuclear DNA for the sequencing libraries, nuclear DNA was isolated from leaves of *P. cablin*. DNA libraries with insert sizes of 180 bp and 500 bp were sequenced using the Illumina HiSeq 2500 platform, generating ~52.62 Gb and 10.88 Gb, respectively (Supplementary Table S1). A total of >43 Gb of clean bases were obtained after removing PCR duplicates, low-quality reads, adaptor sequences and contaminating reads (Supplementary Table S1).

Although organelle genomes are considerably smaller than the nuclear genome, incorrect nuclear DNA extraction can lead to a high percentage of organelle DNA in sequencing libraries, which can dramatically decrease the validity of the data and hinder genome assembly. Therefore, clean reads were aligned to organelle sequences (Supplementary Table S2) using SOAP2^25^. Only 2.91% of the reads aligned (Supplementary Table S3), indicating a low percentage of organelle DNA in our samples. Contamination from microorganisms or other species can also decrease the proportion of valid data. To determine whether there were sequences from other species in the clean data, 10,000 clean reads were randomly selected to align to the National Center for Biotechnology Information (NCBI) nucleotide (NT) database. Most reads (98.36%) aligned successfully to a particular species, and no significant bias for a plant species, and the only significant bias for non-plant species was *Dictyostelium discoideum* (4.74%, Supplementary Table S4), indicating that DNA contamination was inconspicuous.

### Genomic characteristics

**Figure 1:**
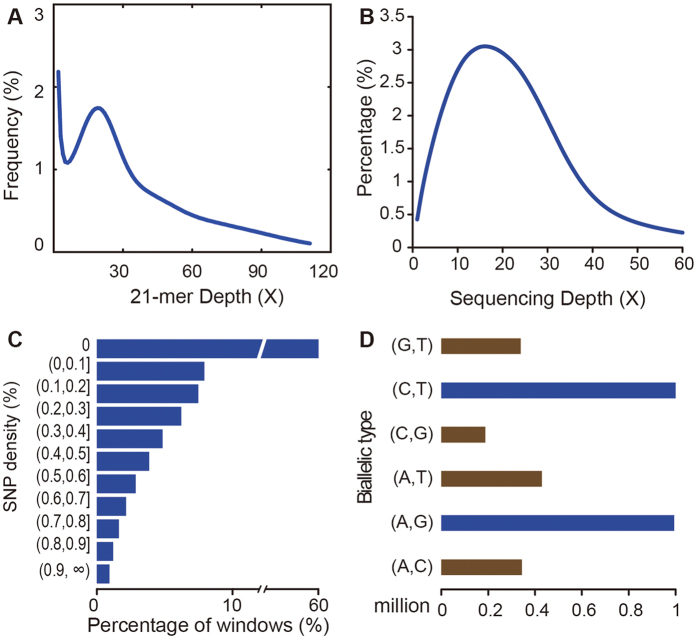
Distribution pattern of 21-mer, sequencing depth, heterozygosity rate and biallelic SNPs. (**A**) 21-mer distribution. The *y*-axis represents the frequency at a given depth divided by the total frequency of all depths. (**B**) Sequencing depth distribution. The *y*-axis is the proportion of the base number at each sequencing depth divided by the total sequenced bases. (**C**) Heterozygous SNP density distribution. Heterozygous SNPs from patchouli diploid genomic data were identified. Non-overlapping 10-kb windows were chosen, and the heterozygosity density was calculated. (**D**) Biallelic SNP distribution. Blue, transition; brown, transversion.

Prior to *de novo* assembly of the genome, we were able to obtain some key genomic features, such as GC content, genome size, and repeat content and heterozygosity rates. These characteristics are vital prior knowledge to guide the sequencing approach and to optimize parameters for the assembly process. Examining the distribution of distinct K-mers is one way to inspect such features. As shown in Fig. 1A, the main peak was at a sequencing depth of 19, and there was no small peak at the depth of 9–10, indicating that the heterozygosity rate of patchouli is relatively low. However, the distribution displays a fat tail, which indicates that this genome contains a high proportion of repeats (Fig. 1A). Indeed, quantitative results from the ALLPATH-LG assembler^26^ also support these conclusions. The patchouli genome was estimated to be ~1576 Mb, and the estimated percentage of repetitive sequences was as high as 72.6%, which is expected when considering the large genome size. The estimated heterozygosity of the reads was 0.46%, a medium level in plants.

### Genome assembly

The clean reads were *de novo* assembled using the de Bruijn graph-based SOAPdenovo2 assembler. Multiply K-mer sizes, including 33, 43, 53, and 63, were examined using default parameters. To obtain the highest number of complete genes, the assembled sequences generated with K = 63 were chosen for the following analyses, as the N50 length was longer than those from other K-mer sizes. The resultant scaffolds were then subjected to gap-filling with the Illumina paired-end (PE) reads by GapCloser, scaffolds shorter than 200 bases or matched to microorganism genomes were removed, and the remaining scaffolds were designated as PCAB_r1.0.

PCAB_r1.0 contains 1,608,748 scaffolds of 1150 Mb total, with contig and scaffold N50 sizes of 416 bp and 1112 bp, respectively (Table 1). Approximately 50% of the total sequence was covered by 238,223 scaffolds of >1112 bp, with the largest scaffold spanning 73 kb. Our assembly is ~73% of the predicted genome size (1576 Mb). This is expected because of the abundant repetitive sequences in the patchouli genome, as it has been demonstrated that assembled genome size can be inflated when there is high repeat content and/or heterozygosity^27^. This percentage is quite similar in the Lamiales relative sesame (*Sesamum indicum* L., 274 Mb relative to an estimated 357 Mb)^28^, although the assembled patchouli genome is much larger.

To assess the quality of the assembly, we aligned all the genome reads to the assembly, and then homozygous single nucleotide polymorphisms (SNPs) were called. Approximately 75.28% of reads were mapped, suggesting a high degree of representation of the patchouli genome in our assembly. Analysis of single base-sequencing coverage (Fig. 1B) revealed a median sequencing depth of 21 and that >95% of the assembly had a coverage >5. The estimated sequencing error or potential misassembly rate was <4 bp per 100 kb, as reflected by homozygous SNPs.

### Heterozygosity rate

Analysis of whole-genome sequences can provide a general overview of the total genetic variation in a species. In this study we identified 3,147,333 heterozygous SNPs and 490,407 insertions and deletions (InDels, length ≤10 bp) in the patchouli genome. This gave an estimated heterozygosity rate (mean per-nucleotide heterozygosity) of 0.274%, revealing relatively high nucleotide diversity. The distributions of SNP density in non-overlapping 10 kb windows revealed a single peak (Fig. 1C), in which the SNP density is similar to the estimated heterozygosity rate. Among these ~3.2 million allelic sites, only 1267 SNPs are multi-allelic. The distribution of types of biallelic sites (Fig. 1D) revealed a high percentage of purine-purine and pyridine-pyridine types. This is not unexpected considering the higher probability of transition than transversion.

### Repeat analysis

Transposable elements (TEs) are a major component of plant genomes and play an important role in plant genome evolution. Comprehensive annotation showed that repeat elements in patchouli make up >673 Mb (58.55% of the assembly; Table 2). This percentage is lower than that estimated by ALLPATH-LG (72.6%). However, these two estimations are quite similar when considering that >461 Mb of the unassembled sequences are generally highly repetitive. The high proportion of repeats is consistent with the fat tail in Fig. 1A and explains, at least in part, the large genome size of patchouli. When compared with closely related species, the repeat content of patchouli is higher than that of sesame (28.5%)^28^, grapevine (52.2%)^29^, and potato (54.5%)^30^ but lower than that of tomato (63.2%)^31^ (Table 2 and Supplementary Table S5). As observed in other sequenced genomes, long terminal repeats (LTRs) make up the majority (22.63%) of repeat sequences in patchouli. A high percentage of unclassified TEs (22.40%, Table 2) indicated a high percentage of patchouli-specific TE categories. For the two major classes of LTRs, the proportion of Copia in patchouli (7.13%) is comparable to that in sesame (7.3%), grapevine (5.16%), and tomato (6.3%) but higher than that in potato (3.8%), whereas the percentage of Gypsy (14.95%) is comparable to that in potato (15.2%) and tomato (19.7%) and much higher than that in sesame (6.6%) and grapevine (3.7%)^28^,^29^,^30^,^31^.

**Figure 2:**
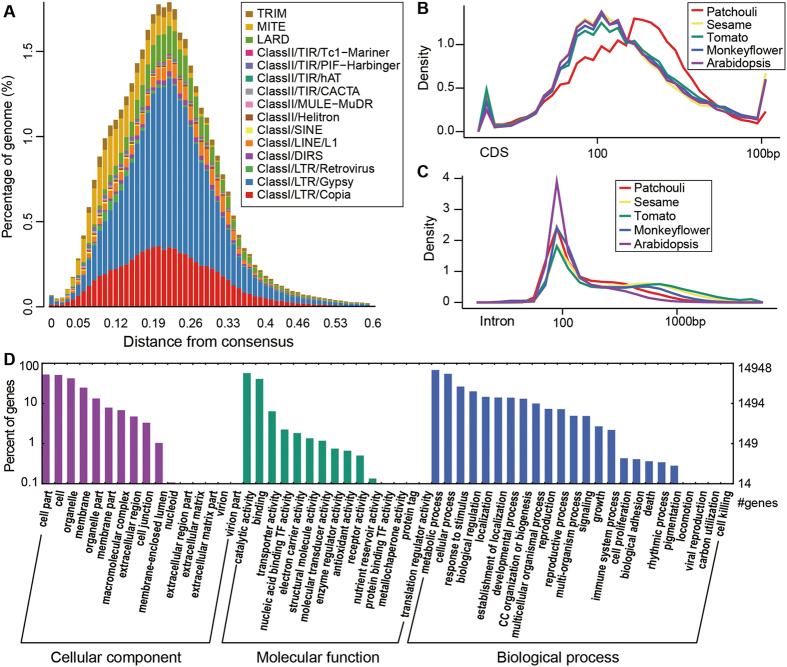
Genome annotation. (**A**) Divergence distribution of TEs in the patchouli genome. (**B**) Distribution of CDS length. The axis are log-10 transferred. (**C**) Distribution of intron length. The axis are log-10 transferred. (**D**) GO annotations of patchouli genes.

Two older TE bursts were identified in the patchouli genome (genetic distances of ~0.10–0.14 and ~0.20–0.24 from the consensus; Fig. 2A), and no recent bursts were observed, indicating the accumulation of many older LTRs and many fewer recent LTRs. These distributions reflect a steady-state stochastic birth/death model for the dynamics of LTR accumulation and activity^30^. Unlike what has been observed in sesame, the divergence of Gypsy in patchouli shows a normal distribution (Fig. 2A), indicating that this species had experienced explosive accumulation or activity, similar to what has been found in tomato and potato^28^.

### Genes in patchouli

A total of 45,020 genes (average length, 431 bases; average GC content, 51.2%) were predicted in PCAB_r1.0 by Augustus^32^ with a training set from *Arabidopsis thaliana*. Among the predicted genes, 25,801 (average length, 494 bases; average GC content, 51.8%) were predicted to be multi-exon genes. To avoid potential contamination by TE-related proteins, the predicted patchouli proteome was compared to the protein (BLASTP^33^) and translated nucleotide (TBLASTN^33^) sequences in RepBase^34^. Nearly all of the predicted genes were found to be intrinsic, and only 33 were TE-related genes, suggesting efficient identification of TEs.

The number of predicted protein-coding genes in patchouli is much higher than in its Lamiaceae relative sesame (27,148)^28^ and Lamiales relative monkeyflowers (26,718) (http://phytozome.jgi.doe.gov/). Considering the fragmental character of our genome assembly and low average length of genes, the gene number in patchouli may be overestimated. Improving the sequencing depth and assembly quality of the patchouli genome and constructing a prediction model based on full-length cDNA sequences obtained from RNA sequencing will improve the quality of gene annotation. Nevertheless, with this assembly we could explore patchouli genes at the exon and intron level (Fig. 2B,C). The coding sequences (CDS) of patchouli exons are longer than those from another three asterid species and from *A. thaliana* (Fig. 2B), whereas the distribution pattern of introns is similar to that of other species (Fig. 2C).

Among the predicted patchouli genes, 25,842 (57.50%) were annotated in functional databases (Table 3), including the NCBI non-redundant (NR) and Clusters of Orthologous Groups (COG) databases and the Kyoto Encyclopedia of Genes and Genomes (KEGG) and Swiss-Prot databases. As shown in Table 3, the NR database has the highest annotation rate (25,274; 56.14%), whereas the KEGG has the lowest (4820; 10.71%). Among the predicted patchouli genes, 17,726 showed similarities to protein-encoding sequences in the Swiss-Prot database. In parallel, a total of 6513 putative genes were classified into COG functional categories. The protein-coding genes were also classified based on gene ontology (GO) by GO-Slim analysis in Blast2GO^35^ with the NR hits, from which 14,949 predicted genes were assigned to at least one GO term (Table 3). The number of genes classified into each of the molecular function, cellular component, and biological process categories are summarized in Fig. 2D. The most abundant categories in molecular function were ‘catalytic activity’ and ‘binding’, whereas ‘metabolic process’ and ‘cellular process’ were the most abundant categories in biological process (Fig. 2D).

### Gene families identification

**Figure 3:**
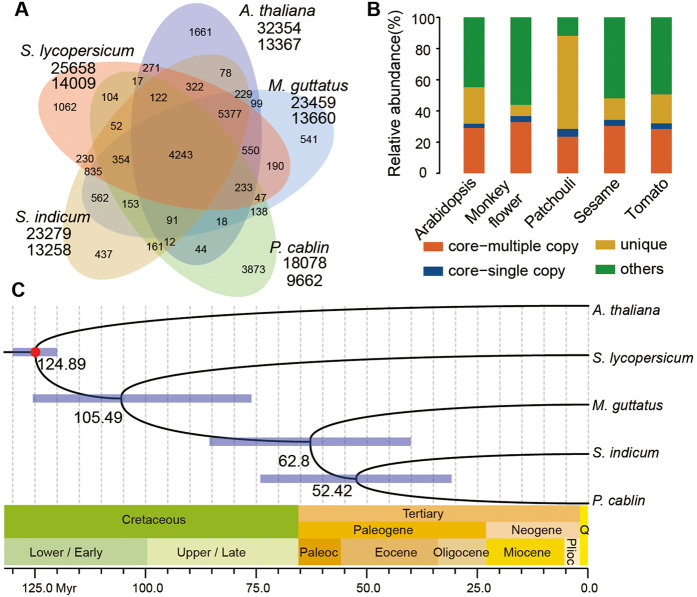
Genome evolution. (**A**) Venn diagram of gene families from five related plant species. (**B**) Spinogram depicting the composition of different categories of gene families. (**C**) Divergence time estimation. The node bars indicate 95% posterior probability intervals. The red dots correspond to calibration points, and the specific calibration time is indicated in the Methods. Plioc, Pliocene; Q, Quaternary.

Predicted protein sequences of patchouli and the complete protein sequence sets of another four plant species, *Solanum lycopersicum*, *A. thaliana*, *S. indicum*, and *Mimulus guttatus*, were binned into 22,106 gene families by OrthoMCL v2.0.9^36^ following self–self–comparisons with the BLASTP program (Table 4). The number of genes in common gene families (in which all five species are represented) from patchouli is smaller than in sesame, monkey flower and tomato (Table 4). However, the average number of genes per gene family of patchouli is comparable to the other species and the number of unique gene families and the number of genes in unique gene families of patchouli are much larger (Table 4). These results underscore that the patchouli gene set is incomplete and fragmented, as we could not get all of the full-length genes from a survey genome. A total of 4,243 gene families are shared by all the five species (Fig. 3A,B), and within these gene families, 923 genes are single copy, meaning that only one orthologous gene exists in each gene family in each species, making them suitable for phylogenetic inference and divergence time estimation.

### Phylogeny inference and divergence time estimation

To maximize the information content of our sequences, multiple protein and corresponding CDS alignments of the 923 single-copy gene families retrieved from OrthoMCL were concatenated into a single supergene for each species using a custom Perl script. The CDS alignments were then subjected to phylogenetic analyses with Bayesian inference implemented in MrBayes^37^ using the GTR+Ι+Γ substitution model. The Bayesian phylogenies obtained were consistent with the maximum likelihood tree and the well-recognized Angiosperm Phylogeny Group III classification system^38^ (Fig. 3C).

Considering that the evolutionary rates are vastly different at the different codon positions, the three codon positions of the supergene concatenated from the 923 single-copy gene families were treated as three different partitions. In the combination analysis of multi-partitions by the MCMCTree program in the PAML4.7 package, the substitution model was used, but different parameters were assigned and estimated for each partition. Moreover, because evolutionary rates have also been observed to vary among species, the clock model with independent rates among lineages specified by a log-normal probability distribution was adopted.

All of the times on nodes in the divergence tree (Fig. 3C) were well-matched to data deposited in TIMETREE^39^, a public knowledge-base of divergence times among organisms, demonstrating the high reliability of this molecular clock dating strategy. As shown in Fig. 3C, the divergence between patchouli and sesame appears to have occurred ~52.42 (30.90–74.10) Mya, during the Eocene. The Phrymaceae (monkey flowers) and Lamiaceae-Pedaliaceae (patchouli-sesame) shared a most recent common ancestor during the Paleocene, ~62.80 (40.1–85.6) Mya. A previous study revealed a recent whole-genome duplication (WGD) event in sesame^28^ that occurred independently of a parallel triplication event in the tomato–potato lineage during the same period. The whole-genome triplication that occurred in tomato^31^ and was also evident in potato^30^ was estimated at 71 (±19.4) Mya based on the Ks of paralogous genes, which is earlier than the splitting of patchouli and sesame (~52 Mya), therefore implying that both patchouli and sesame experienced the recent WGD. The complete patchouli genome sequence and additional high-quality Lamiaceae genomes will help to validate the phylogenetic dating of the recent WGD events that occurred before the divergence of Lamiaceae species.

### Genes involved in sesquiterpenes biosynthesis

**Figure 4:**
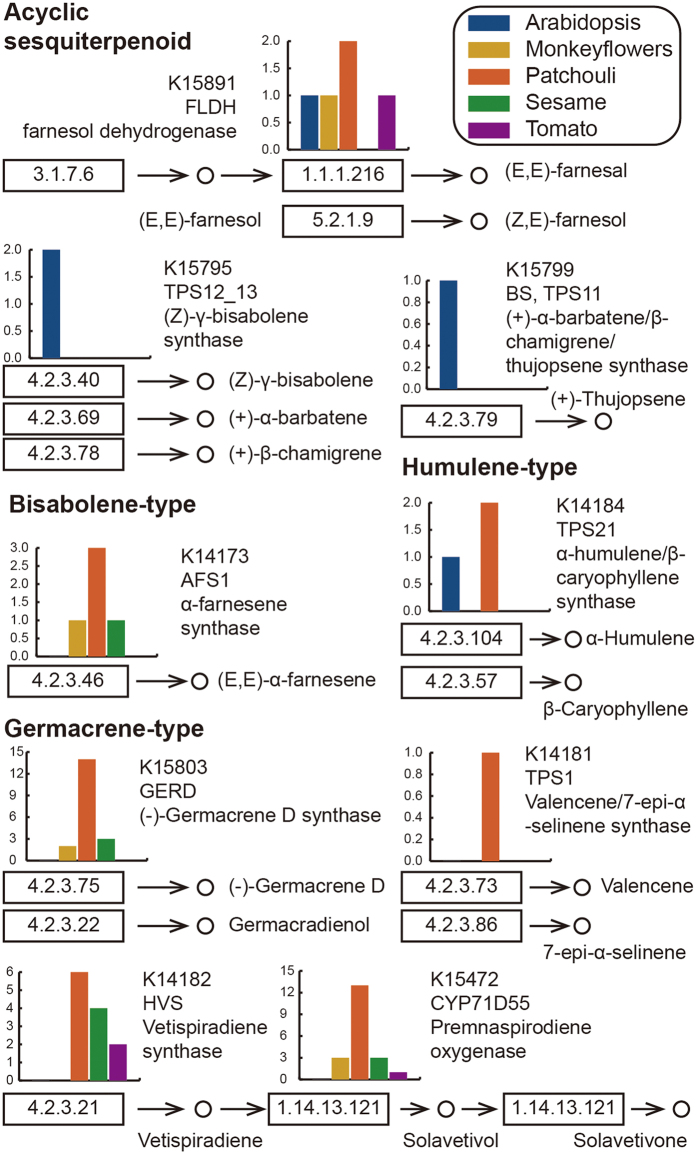
Sesquiterpenoid biosynthesis pathway. Circles indicate chemical components; rectangles are enzymes, with the EC numbers given; bar plots show the gene copy number in each species.

To elucidate sesquiterpenoid biosynthesis in patchouli, the sesquiterpenoid biosynthesis pathway–related data were first extracted from the sesquiterpenoid and triterpenoid biosynthesis reference pathway (map00909) in KEGG^40^. The resultant sesquiterpenoid biosynthesis reference pathway contains 43 unique KEGG orthology (KO) entries (Supplementary Table S6). Protein-coding genes in patchouli were then mapped onto the KEGG metabolic pathways using the KEGG automated annotation server (KAAS)^40^ along with the genes from several other plant species, including Arabidopsis, tomato, sesame, and monkey flower. Finally, genes annotated with KO entries were mapped onto the sesquiterpenoid biosynthesis reference pathway (Supplementary Table S7). Of the 43 KO entries in the pathway, patchouli contained the most (seven KO entries), whereas monkey flower, sesame and Arabidopsis overlapped with four KO entries each, and tomato covered only three (Fig. 4). As shown in Fig. 4, genes in Arabidopsis are biased toward acyclic sesquiterpenoid biosynthesis, whereas genes in patchouli are involved in the biosynthesis of nearly all types of sesquiterpenoids. In addition to the coverage of the reference pathway, the gene copy numbers in patchouli were also higher than those in other species (Fig. 4).

## Methods

### Plant material and DNA extraction

Samples were collected from Fenglai Village, Yangchun City, Guangdong Province, China. High-quality genomic DNA was extracted from the leaves of *P. cablin* using an improved CTAB method^41^. The modified CTAB extraction buffer included 0.1 M Tris-HCl, 0.02 M EDTA, 1.4 M NaCl, 3% (w/v) CTAB and 5% (w/v) PVP K40, and -mercaptoethanol was added to the CTAB extraction buffer to ensure DNA integrity and quality. RNase A and proteinase K were used to remove RNA and protein contamination, respectively.

### Genome sequencing and data filtering

Two PE Illumina genomic DNA libraries (insert sizes of 180 and 500 bp, respectively) were then constructed. The libraries were sequenced (100 bp PE) on an Illumina HiSeq 2500 system. All raw sequencing files have been submitted to the NCBI Sequence Read Archive under accession number PRJNA295004. FASTQ files from all sequencing runs were imported and subjected to quality control. Clean reads were obtained by removing PCR duplicates, low-quality reads, adaptor sequences and contaminating reads of bacterial or viral origin. Additionally, sequence errors were corrected based on the K-mer frequency.

### Quality assessment of sequencing data

To determine whether the data were contaminated by other species, we randomly selected 10,000 clean reads from the library with insert size of 180 bp, and these reads were aligned to the NT library in the NCBI database using the BLAST software (parameters: *-b 100 -v 100 -p blastn -e 1e-05 -F F*). We determined the species of the highest scoring BLAST hit for each read. To evaluate the percentage of contaminating reads from organelle genomes in the data, Illumina PE reads were aligned to the organelle sequences using SOAP2^25^ (parameters: *-m 100 -x 280 -p 10*). Organelle sequences included sequences from chloroplasts (*P. cablin*, *Salvia miltiorrhiza*, *S. indicum*, and *Vitis vinifera*) and mitochondria (*S. miltiorrhiza* and *V. vinifera*) (Supplementary Table S3).

### Genomic characteristics, assembly, assessment and heterozygosity rate

Before genome assembly, genomic characteristics, including GC content, genome size, potential repeat content and heterozygosity rates, were estimated by ALLPATH-LG^26^. SOAPdenovo2^42^ was adopted for assembly of the clean Illumina PE reads. K-mer sizes of 33, 43, 53, and 63 were examined using default parameters, and the optimal k-mer size (k = 63) was selected based on the N50 length in each k-mer size. The resultant scaffolds were subjected to gap-filling with the Illumina PE reads by GapCloser 1.10 (P = 31) (http://soap.genomics.org.cn/soapdenovo.html), and scaffolds >200 bp were selected and designated PCAB_r1.0.

To assess the degree of heterozygosity, sequencing errors, and/or potential assembly errors, we aligned all of the genome reads to the assembly using the BWA-MEM algorithm^43^. The depth of each base was calculated by the ‘depth’ module of SAMTOOLS^44^ with default settings. SNPs/InDels were called by the SAMTOOLS-BCFTOOLS pipeline^44^ (parameters: *samtools mpileup -q 1 -C 50 -g -t DP*, *SP -m 2*), followed by variants filtering steps implemented by vcfutils.pl (parameters: *vcfutils.pl varFilter -Q 20 -d 5 -D 250 -w 5 -W 10*).

### Repetitive sequence analysis

The patchouli *de novo* repeat library was constructed using four complementary programs, LTR_finder^45^, RepeatScout^46^, PILER-DF^47^, and MITE-Hunter^48^, followed by an additional classification step implemented by PASTEClassifier^49^. The resulting library sequences with their classification information were integrated with RepBase^34^ to generate the final library, which was used to run RepeatMasker^50^ on the assembled scaffolds.

### Gene prediction and annotation

From the repeat-masked scaffolds >1000 bp in PCAB_r1.0, genes were predicted by Augustus^32^ with a training set of *A. thaliana* (TAIR10). The parameters used were: species = arabidopsis, genemodel = partial, protein = on, introns = on, start = on, stop = on, cds = on, codingseq = on, alternatives-from-evidence = true, alternatives-from-sampling = true, gff3 = on, UTR = on. The predicted genes were classified into four categories, intrinsic (with start and stop codons), partial (without start and/or stop codons), pseudo (with in-frame stop codons), and short genes (encoding <50 amino acids).

### Gene functional annotation

Annotation of the predicted genes was performed by carrying out a BLAST analysis of patchouli sequences against a number of protein sequence databases, including the NCBI NR and COG^51^ databases, Swiss-Prot^52^, and KEGG^40^, using an E-value cutoff of 1e-5. KEGG pathways were retrieved from the KEGG web server (http://www.genome.jp/kegg/)^53^. The output of the KEGG analysis includes KO assignments and KEGG pathways that are populated with the KO assignments. Domain-based alignments were carried out against the COG database^51^ (http://www.ncbi.nlm.nih.gov/COG/) with a cut-off E-value of ≤1e-5. The resulting NR BLASTP hits were processed by the Blast2GO software^35^ to retrieve associated GO terms with an E-value ≤1e-5 describing biological processes, molecular functions, and cellular components^54^.

### Gene families identification

The predicted proteins of *P. cablin* were pooled into a protein database with sequences >50 amino acids from another four plant species, *S. lycopersicum* (Sol Genomics Network ITAG2.3 Release), *A. thaliana* (TAIR release 10), *S. indicum*, and *M. guttatus*. For those having spliced variants, only the variants with the longest protein sequences were used. Pairwise sequence similarities between all protein sequences were calculated using BLASTP^33^ with an E-value cutoff of 1e-05, and hits with identity <30% and coverage <30% were removed. Then, ORTHOMCL v2.0.9^36^ was used to perform a Markov clustering algorithm to define the cluster structure, with a default inflation value (-I) of 1.5.

### Phylogenetic tree reconstruction

Single-copy gene families were retrieved from the ORTHOMCL results and used for the following phylogenetic maximum likelihood and Bayesian tree reconstruction steps. First, the proteins in each family were aligned using MUSCLE v3.8.31^55^ with default parameters. For Bayesian tree reconstruction, the CDS alignments of single-copy gene families were back-translated from the corresponding protein alignments and concatenated into a single supergene for each species using a custom Perl script. These supergenes were then subjected to phylogenetic analyses with MrBayes^37^ using the GTR+Ι+Γ substitution model. For maximum likelihood tree reconstruction, the amino acid alignments of each gene family were assembled into supergenes, and these supergenes were then subjected to MEGA^56^ using the maximum likelihood method. The parameters used in the tree construction were the JTT model with gamma-distributed rates and 1000 bootstraps.

### Divergence time estimation

The CDS alignments of each gene family obtained in the Bayesian tree reconstruction step were separated into three partitions, corresponding to each one of the three codon positions in the CDS. The three supermatrices corresponding to each codon position were then separately assembled into ‘supergenes’ using a custom Perl script. Divergence times were estimated under a relaxed clock model using the MCMCTree program in the PAML4.7 package^57^. The “Independent rates model (clock = 2)” and “JC69” model in MCMCTree were used in our calculation. The MCMC process is run for 4,000,000 iterations after a burn-in of 2,000,000 iterations. Other parameters used the default settings of MCMCTree. To check the robustness of the results, we ran the MCMCTree analysis twice and obtained similar results, and a chronogram was produced using FigTree v1.4.0 (http://tree.bio.ed.ac.uk/) with the first run. We selected 120 and 130 Mya as the lower and upper boundaries for the Eurosid–Asterid split (Arabidopsis–tomato)^39^.

## Additional Information

**How to cite this article**: He, Y. *et al.* Survey of the genome of *Pogostemon cablin* provides insights into its evolutionary history and sesquiterpenoid biosynthesis. *Sci. Rep.* **6**, 26405; doi: 10.1038/srep26405 (2016).

## Supplementary Material

Supplementary Information

Supplementary Tables

## Figures and Tables

**Table 1:** Statistics of the patchouli genome assembly PCAB_r1.0.

Characteristic	Value
Total length (bp)	1,150,613,626
Total (ATGC)	1,079,532,052
Number of sequences	1,608,748
Number of sequences ≥2 kb	100,319
Average sequence length (bp)	715
Maximum sequence length (bp)	73,268
Scaffold N50 length (bp)	1112
Contig N50 length (bp)	416
GC% (GC/ATGC)	34.9

**Table 2:** Statistics of the repetitive sequences in the patchouli genome.

Class	Counts	Length (bp)	Percentage
ClassI/DIRS	10,816	6,645,587	0.58%
ClassI/LINE	51,614	20,311,897	1.77%
ClassI/LTR	432,245	260,410,857	22.63%
ClassI/PLE	123	7849	0.00%
ClassI/SINE	10,418	1,345,276	0.12%
ClassI/Unknown	20,139	6,742,968	0.59%
ClassII/Crypton	23	1355	0.00%
ClassII/Helitron	11,299	3,267,126	0.28%
ClassII/Maverick	1603	809,213	0.07%
ClassII/TIR	30,624	8,532,182	0.74%
ClassII/Unknown	6798	1,634,777	0.14%
LARD	71,075	26,548,156	2.31%
MITE	242,179	33,204,936	2.89%
PotentialHostGene	7,603	2,060,266	0.18%
SSR	477,463	27,799,796	2.42%
TRIM	35,462	16,572,802	1.44%
Unknown	1,466,155	257,768,490	22.40%
Total	2,875,639	673,663,533	58.55%

DIRS, *Dictyostelium* intermediate repeat sequence;

LINE, long interspersed nuclear element;

LTR, long terminal repeat;

PLE, Penelope-like elements;

SINE, short interspersed nuclear element;

TIR, terminal inverted repeat;

LARD, large retrotransposon derivative;

MITE, miniature inverted-repeat transposable element;

SSR, simple sequence repeat;

TRIM, terminal repeat retrotransposon in miniature.

**Table 3:** Functional annotation of genes from the patchouli genome.

Functional database	# of annotated genes	Percentage
COG	6513	14.47%
GO	14,949	33.21%
KEGG	4820	10.71%
Swiss**-**Prot	17,726	39.37%
NR	25,274	56.14%
Total	25,842	57.40%

**Table 4:** Summary of the gene families among five related plant species.

Species	Genes in families	Families	Unique families	Genes in unique families	Genes in common families	Genes per families
Patchouli	18,078	9662	3873	10,770	5176	1.871
Sesame	23,279	13,258	437	3195	8002	1.756
Monkey flower	23,459	13,660	541	1675	8622	1.717
Tomato	25,658	14,009	1062	4770	8220	1.832
Arabidopsis	32,354	13,367	1661	7538	10,330	2.420

Common families are the families that all species presented.

Unique families are the families that only particular species presented.
