# Supplementary material for: Survey of the genome of Pogostemon cablin provides insights into its evolutionary history and sesquiterpenoid biosynthesis
Source: Sci Rep. 2016 May 20;6:26405. doi: 10.1038/srep26405 (PMC4873823; doi:10.1038/srep26405)
Supplement: Supplementary Information [file srep26405-s1.pdf]

Survey of the genome of *Pogostemon cablin* provides insights into its evolutionary history and sesquiterpenoid biosynthesis

Yang He[1,*], Hongtao Xiao[2,3,*], Cao Deng[4], Liang Xiong[1], Hu Nie[4] & Cheng Peng[1]

[1] State Key Laboratory Breeding Base of Systematic Research, Development and Utilization of Chinese Medicine Resources, Chengdu University of Traditional Chinese Medicine, Chengdu 610075, China

[2] School of Medicine, University of Electronic Science and Technology of China, Chengdu 610072, China

[3] Department of Pharmacy, Hospital of the University of Electronic Science and Technology of China and Sichuan Provincial People's Hospital, Chengdu 610072, China

[4] DNA Stories Bioinformatics Center, Chengdu 610065, China

*These authors contributed equally to this work.

Correspondence and requests for materials should be addressed to C.P. (email: tcmpengcheng@163.com)

1. Supplementary Tables S1-S7 were submitted as ‘Supplementary Dataset’ in the manuscript tracking system:

Table S1. Statistics of the sequencing data.

Table S2. Length of organelle genomes of selected species.

Table S3. Statistics of reads mapping to plastid sequences.

Table S4. Statistics of reads mapping to the NCBI NT database.

Table S5. Statistics of sub-categories of repetitive sequences in the patchouli genome.

Table S6. Sesquiterpenoid biosynthesis reference pathway.

Table S7. Genes annotated to the sesquiterpenoid biosynthesis reference pathway.

2. Supplementary files related to gene annotation, including gff3 file and fasta files of coding sequences and protein sequences were submitted to https://github.com/brentcaodeng/patchouli_genome_survey/tree/master/01.Annotation
